# Minimally invasive aspiration of thrombi and masses in left-sided cardiac chambers: a comprehensive literature review

**DOI:** 10.3389/fcvm.2025.1597893

**Published:** 2025-11-12

**Authors:** Ziyad Gunga, Augustin Rigollot, Margaux Wolff, David Meier, Eric Eeckhout, Valentina Rancati, Zied Ltaief, Mario Verdugo-Merchese, Olivier Muller, Matthias Kirsch

**Affiliations:** 1 Department of Cardiovascular Surgery, Lausanne University Hospital, Lausanne, Switzerland; 2 Department of Cardiology, Lausanne University Hospital, Lausanne, Switzerland; 3 Lausanne University, UNIL, Lausanne, Switzerland; 4 Department of Anesthesiology, Lausanne University Hospital, Lausanne, Switzerland

**Keywords:** thromboaspiration, left-sided masses, AngioVac, novel procedures, innovation, neuroprotection device

## Abstract

The management of large intracardiac masses, such as thrombi, tumors, or endocarditic vegetations, presents significant challenges due to their friable nature and the risks of embolization or hemodynamic compromise. While surgical removal remains the gold standard, it is often contraindicated in high-risk patients. Minimally invasive techniques, particularly thromboaspiration, offer a promising alternative, especially for left-sided cardiac chambers where systemic circulation and delicate anatomy heighten procedural risks. This review analyzes the evolving role of thromboaspiration for left-sided cardiac masses, focusing on 24 cases from 14 studies published between 2014 and 2024. Most cases utilized the AngioVac® system, with others employing devices such as Lasso®, Occlutech®, and Amplatz® systems. Neuroprotection was implemented in 79% of cases, primarily using Sentinel® devices. Access was predominantly transseptal, though transapical, transcaval, and femoral routes were also utilized. Notably, 88% of procedures were performed without ECMO support. The results highlight a high success rate (92%) in mass removal with minimal complications, although potential publication bias must be acknowledged. This success underscores thromboaspiration's viability not only for patients unfit for surgery but also for those with intracavitary or pedunculated thrombi. Thromboaspiration represents a less invasive, effective solution for managing left-sided cardiac masses, expanding its applicability beyond right-sided cases. This review emphasizes the need for further studies to establish standardized protocols and encourage broader adoption of this innovative technique in clinical practice.

## 1 Introduction

The presence of large thrombi, tumor, or endocarditic vegetation within the cardiac chambers or attached to valves poses a significant clinical challenge. These masses, often resistant to pharmacological or conventional treatments, demand careful management due to their friable nature and the associated risks of embolization or hemodynamic compromise (1). While intravenous or intracardiac thrombolytics have shown limited success, surgical removal remains the gold standard, particularly in cases where the mass affects valvular function or poses an immediate threat (2). However, surgery is not always an option, especially in patients deemed unfit for invasive procedures due to comorbidities, age, or previous surgeries.

For these patients, minimally invasive approaches offer an increasingly viable alternative, addressing their need for intervention while minimizing the risks associated with conventional cardiac surgery. Recently, guidelines have integrated right-sided cardiac aspiration into the therapeutic armamentarium, reflecting the success of vacuum-assisted devices in debulking obstructive masses in the right heart and pulmonary vasculature (3). The next frontier, now gaining traction, is applying these techniques to the left-sided cardiac chambers, where the risks are inherently higher due to the systemic circulation and more delicate anatomy (4).

Although traditionally limited to patients with severe contraindications for surgery, left-sided thromboaspiration is evolving as a key treatment modality not just for those unfit for surgery, but also for cases where thrombi are intracavitary or pedunculated on valves. This approach offers a less invasive option, potentially preventing life-threatening complications such as embolization or valvular dysfunction, while maintaining procedural safety and efficacy.

Hence, the surge in innovative percutaneous or minimally invasive aspiration techniques for managing left sided intracardiac mass marks a significant advancement in the field. These methods, building on the successes seen in right-sided cases (5), are rapidly gaining recognition in many centers. Hence, we find it essential to explore the existing literature and provide a thorough review of this technique, with the objective of broadening its understanding and facilitating its adoption in clinical practice.

## 2 Methodology

**Figure 1:**
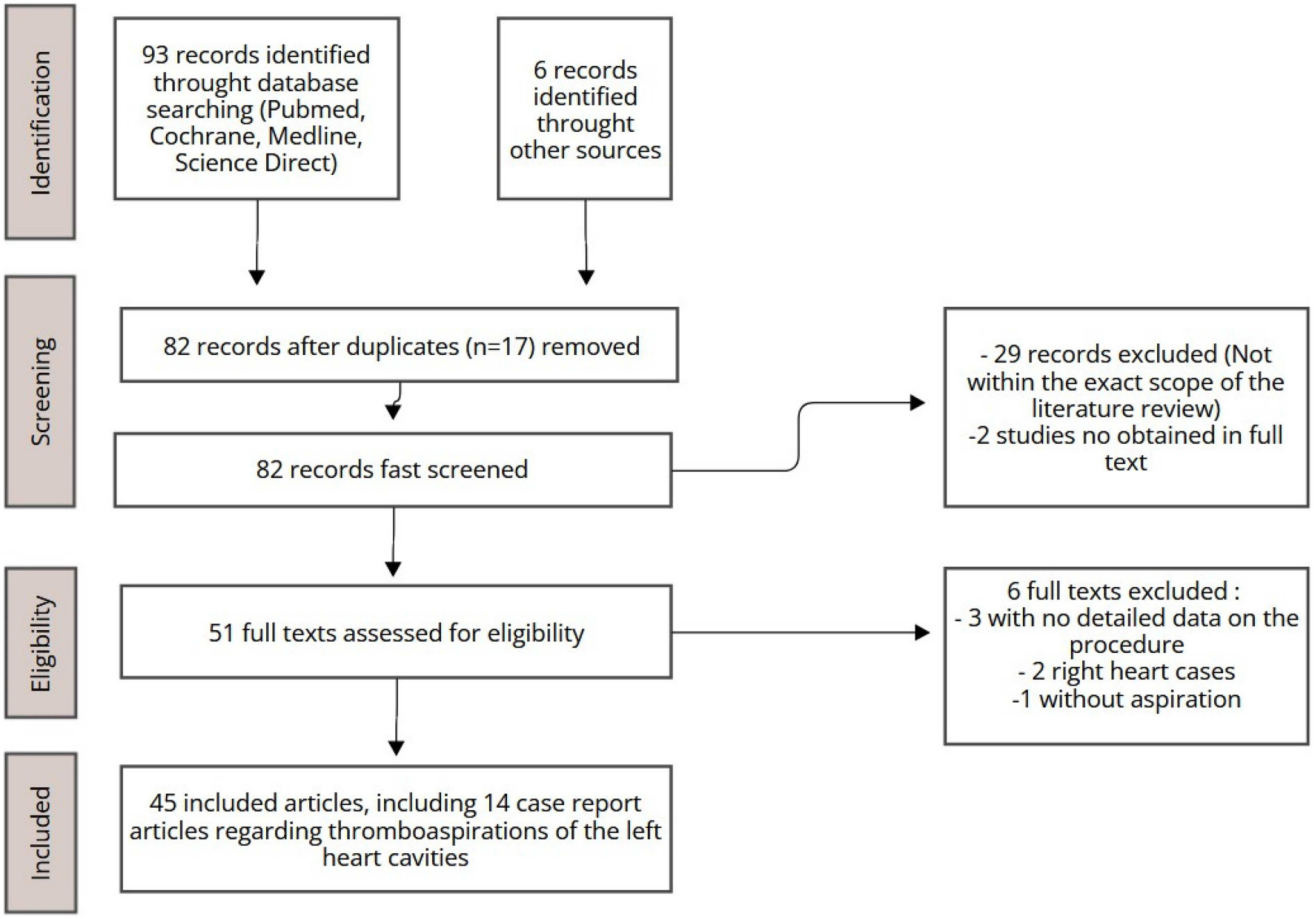
Study selection process.

The literature review was carried out using the PubMed, Medline, ScienceDirect, and Cochrane databases. Initially, 99 articles were identified, predominantly covering the period from 2014 to 2024, with a few seminal works included as exceptions (Figure 1). After excluding irrelevant studies and duplicates, 45 articles were deemed relevant and selected for qualitative analysis. Emphasis was placed on recent case reports focusing on aspiration within the left heart chambers, ensuring an up- to-date exploration of contemporary practices.

## 3 Results

In recent years, thromboaspiration has predominantly been employed in right heart chambers, where it was initially used for the retrieval of thrombi (6–8) However, growing evidence now supports its application in the left heart chambers. In this review, we present 24 cases from 14 published articles (see Table 1), including 10 cases from the series by Qintar et al. The average patient age was 57.7 years, comparable to the 58.3 years reported by Qintar, with a gender distribution of 45% women and 55% men. The majority of patients (20 cases) underwent aspiration using the AngioVac® system (AngioDynamics, Latham, NY, USA), while others were treated with the Lasso® (Biosense Webster Inc., Diamond Bar, CA), Occlutech® (International AB, Helsingborg, Sweden), or Amplatz® Goose Neck Snare systems (Medtronic, Tolochenaz, Switzerland). Neuroprotection devices were used in 19 cases, with the Sentinel® device (Boston Scientific, Marlborough, MA, USA) being most common (15 cases), followed by Triguard® (TriGuard HDH, Keystone Heart, Tampa, FL, USA) (2 cases) and SpiderFX® (Medtronic, Minneapolis, MN) (2 cases). In some instances, no neuroprotection was employed due to incidental thrombus findings or procedural alternatives like carotid clamping. Most cases (21) were performed without ECMO support, with the transseptal route being the most common access approach, followed by transapical, transcaval, and femoral routes. Some cases required a mini-anterior right thoracotomy, while others were managed solely percutaneously. Overall, 22 cases were successful, both in terms of mass removal and complication rates, though potential publication bias in favor of successful outcomes must be considered.

**Table 1:** List of current case reports in the literature emphasizing on left sided cavities mass aspiration. (AA, arterio-arterial; VV, veno-venous).

Source	Age and sex	Location and type of mass	Method used for thromboaspiration	Cerebral protection system (CPS)	Success or failure	Follow-up duration
Bansal et al. (9)	66, Female	Large endocarditic vegetation on the native mitral valve, accompanied by a developing abscess	AngioVac® aspiration through the right femoral vein after dilation. The reinfusion was performed into the left femoral vein with a 17Fr cannula.	Sentinel® bilateral	Success Clinical outcome: remain hemodynmically stable and can underwent hip surgery	NO
Fiocco et al. (10)	57, Female	Native mitral valve endocarditis	AngioVac® Transapical, AA	TriGuard®	Success (mass disappeared, stump left) Clinical outcome: hemodynamically stable, neurological status intact	TTE at one week after intervention, mild mitral regurgitation (MR) and no regrowth. 6 weeks IV antibiotics
Fiocco et al. (10)	54, Male	Mitral and aortic bioprosthesis endocarditis	Transapical AngioVac® system, AA	TriGuard®	Success (no significant residual mass) Stable, no neurological impairment, no ischemia	1 month: TTE: partial detachment of the aortic prosthesis & moderate paravalvular leak, no regrowth 6 months: no leak progression, no vegetation
Frisoli et al. (11)	77, Male	Thrombus on Watchman closure device	AngioVac® transapical, bilateral femoral veinous access	Sentinel® bilateral	Success Clinical outcome: discharged day 2 after the intervention, on warfarin, without complications	1 month follow-up: TEE showed no thrombus
Gerosa et al. (12)	70, Female	Mitral bioprostheses mass later identified as a thrombus	AngioVac®mini- anterior left thoracotomy, transapical access + concomittant ECMO	No	Success Clinical outcome: successfully weaned from ECMO	NO
Gerosa et al. (13)	68, Female	Mass on the mitral valve	AngioVac® AV, trans- atrial with mini- anterior right thoracotomy + bipump concomitant ECMO	N/A	Success Clinical outcome successfully weaned from ECMO seven day later	NO
Gunga et al. (4)	54, Male	Motile mass (18 mm × 11 mm) pedunculated to the mitral bioprosthesis	Occlutech® transapical AV, with mini-anterior left thoracotomy	Sentinel® bilateral	Success Clinical outcome: uneventful, no complication, discharged home after few days	12 months: free of relapse
Kucuk et al. (14)	68, Female	26 mm × 8 mm mass attached to a calcified posterolateral mitral annulus (CMAC) in left atrium	AngioVac® VV, trans- septal	Sentinel® bilateral	Success Clinical outcome: uneventful postprocedure hospital course and discharged home	NO
Lane et al. (15)	57, Male	Large mobile vegetation on the atrial side of the posterior mitral leaflet	AngioVac® AV transeptal acess throught femoral vein + concomitant ECMO	Multimodal neuroprotection: SpiderFX® (2 femoral arteries, one innominate right) + balloon in left subclavian artery	Success 80% Clinical outcome: 4 weeks of inpatient care, no Other embolic events, patient discharged	6 weeks: TTE mild mitral regurgitation, vegetation now longer seen
Latcu et al. (16)	51, Male	Incidental discovering of a hight mobile thrombus in left atria during AF ablation	Double lasso	No	Success Clinical outcome: the patient remained asymptomatic and no embolic physical sign was seen	NO
Patil et al. (17)	22, Male	Left ventricular mass (mobile, pedunculated)	Percutaneous transcatheter snaring and extraction with Amplatz Goose Neck snare	SpiderFX®	Success (mass fully evacuated), Clinical outcome: The patient did not have any clinical evidence of neurological deficit or peripheral embolization	NO, only post-procedure TTE and CMR
Qintar et al. (18)	65, Female	Large left atrial thrombus	AngioVac® AV configuration, transseptal access	Bilateral Sentinel® device	Failure (0% aspirated) Clincial outcome: no complication	7 months follow-up: alive and without complication
Qintar et al. (18)	36, Male	Acute thrombus in the left ventricle/left ventricular outflow tract	AngioVac® AV configuration, transseptal access	Bilateral Sentinel® device	Partial success (20% for left ventricular thrombus part-100% for left ventricular outflow tract part) Clinical outcome: no complication	5 months follow-up: no complication, alive
Qintar et al. (18)	55, Female	Aortic arch thrombus	AngioVac® AV configuration, transcaval approach	Bilateral Sentinel® device	Success (70% aspirated)	4 months: alive and no complication
Qintar et al. (18)	86, Female	Persistent left atrial appendage thrombus	AngioVac® AA configuration, transseptal access	Bilateral Sentinel® device	Success (100% aspirated)	4 months: alive and no complication
Qintar et al. (18)	77, Female	Persistent left atrial appendage thrombus	AngioVac® AA configuration, transseptal access	Bilateral Sentinel® device	Partial success (50% aspirated)	1 month: alive, no complication
Qintar et al. (18)	60, Male	Posterior mitral valve ring thrombus	AngioVac® AA configuration, transseptal access	Bilateral Sentinel® device	Success (70% aspirated)	1 month: alive, no complication
Qintar et al. (18)	40, Male	Aortic arch thrombus	AngioVac® AA configuration, transcaval access	Bilateral Sentinel® device	Success (90% aspirated)	1 month: alive, no complication
Qintar et al. (18)	48, Male	Aortic arch thrombus and abdominal aortic thrombus	AngioVac® AV configuration, femoral artery access	Right-sided Sentinel® with left subclavian 8.0 Armada occlusion balloon	Success (80% aspirated)	16 months: alive, no complication
Qintar et al. (18)	31, Male	Mitral valve vegetation	AngioVac® AA configuration, transseptal access	Bilateral Sentinel® device	Success (90% of the vegetation aspirated)	3 months: alive, no complication
Qintar et al. (18)	43, Male	Mitral valve vegetation	AngioVac® AA configuration, transseptal access	Bilateral Sentinel® device + balloon	Success (100% aspirated)	2 months: alive, no complication
Sorajja et al. (19)	61, Female	Incidental discovering of pedunculated thrombus attached near the distal shaft of the catheter,	Double Lasso®	No, due to incidental discovering	Success for the aspiration The AF ablation was stopped and reprogrammed 2 months later	At 2 months follow-up, no transient ischemic attack or stroke was noted, and a hypercoagulable work-up unremarkable
		during AF ablation				
Tsilimparis et al. (20)	66, Female	Free-floating mural thrombus in the ascending aorta	AngioVac® AV, left subclavian artery access	No device, < 1 min carotids clamping during the intervention as neuroprotection	Success Clinical outcome: uneventful postoperative course, no signs of free thrombus on the post-operative CTA	8 months follow-up, no residual or recurrent thrombus was present on the CTA
Umadat et al. (21)	72, Male	1.4 cm × 1.0 cm left atrial myxoma	AngioVac® VV, trans- septal, FLORIDA Procedure	Sentinel® unilateral	Success Clinical outcome: well tolerated, no complication	NO

### 3.1 Case reports and state of the art techniques utilized

The first documented case of left thromboaspiration in the literature dates back to 2009, reported by Latcu et al. (16) during an atrial fibrillation (AF) ablation. A 51-year-old man underwent radiofrequency ablation, and preoperative transesophageal echocardiography (TEE) revealed no left atrial masses. However, a highly mobile thrombus was discovered during the procedure. After failed attempts to dissolve it with heparin and unsuccessful aspiration using the Lasso® system, a second transseptal sheath was introduced to remove the thrombus by manual aspiration. The patient had no embolic complications, despite the absence of a neuroprotection device.

In 2010, a case report (19) detailed a similar scenario involving a 61-year-old woman undergoing paroxysmal AF ablation. Pre-procedural imaging showed no evidence of thrombus, but during the intervention a thrombus was detected near the catheter's distal tip. Utilizing the two sheaths already in place, the thrombus was aspirated. No neuroprotection device was employed. The patient experienced no complications.

In 2018, a case report (20), described a 66-year-old female patient with a free-floating mural thrombus in the ascending aorta who was deemed surgically unfit because of multiple comorbidities. Thromboaspiration was successfully performed using the AngioVac® system, with an arterio-venous circuit established between the proximal left subclavian artery and the femoral vein for venous reinfusion. Neuroprotection was not employed, but the carotid arteries were clamped for less than a minute during the procedure. The patient had no complications or recurrence of the thrombus post- procedure.

That same year, another case (17) involved a 22-year-old patient who underwent percutaneous extraction of a left ventricular mass. The mass, although non-malignant, was fibrous and inflammatory, located at the apex of the left ventricle. The patient declined conventional surgery, so the mass was aspirated using the Amplatz® Goose Neck device after femoral arterial access was obtained. Neuroprotection was ensured by deploying a SpiderFX device in each carotid artery. The procedure was successful, with no complications or embolic events, and the mass was completely removed.

Gerosa et al. (13) reported a case in 2019 involving a 68-year-old woman with a mitral valve mass, presenting symptoms of dysarthria, right-hand paralysis, and fever. an estimated morbidity-mortality risk exceeding 40%, conventional surgery was deemed too risky. Instead, a minimally invasive thromboaspiration approach was employed. Femoral access established ECMO support with a 21Fr venous and a 23Fr arterial cannula. A right mini-thoracotomy was performed in the second intercostal space, allowing insertion of a 22Fr AngioVac® cannula into the left atrium. The procedure successfully aspirated the mass, confirmed as a thrombus. The patient recovered well and was weaned from ECMO after seven days.

In 2020, Gerosa et al. (12) refined their procedure by modifying the access point. They reported a case involving a 70-year-old patient with mitral and aortic bioprostheses, who presented with a mitral mass. Given the high risk associated with redo surgery, thromboaspiration was performed using the AngioVac® system via transapical access following a left anterior mini-thoracotomy. Hemodynamic support was provided through ECMO and no complications were reported post-procedure.

In 2021, Frisoli et al. (11) reported the case involving a 77-year-old patient with a history of atrial fibrillation (AF), presenting with a thrombus on a Watchman device for left atrial appendage closure, 45 days post-implantation. The thrombus was successfully aspirated using AngioVac® via a transseptal approach after femoral venous access. Neuroprotection was ensured with bilateral Sentinel® devices. Following the procedure, a minimal residual thrombus remained, but the patient did not experience any complications.

The 2022 series of 10 cases by Qintar et al. (18) stands out as the largest reported to date, featuring a well-balanced cohort (50% male, 50% female) with a mean age of 58.3 years—younger than previous studies. Patients, treated between February 2020 and November 2021 in Michigan, primarily presented with recurrent embolic events. Thrombi locations included the left atrium, left atrial appendage, aortic arch, and left ventricle. AngioVac® was used via transseptal (7 cases), transcaval (2 cases), and femoral (1 case) routes, with arterio-venous (AV) and arterio-arterial (AA) circuits employed. Neuroprotection was provided in all cases. The series reported 80% successful aspiration (defined as at least 70% removal) without complications, while two cases of thromboaspiration failure were noted.

In 2023, a series (10) of two cases reported successful evacuation of endocarditic vegetation using the AngioVac® system. In the first case, a 57-year-old woman had a 20 mm vegetation on the native mitral valve, evacuated via transapical access through a mini-thoracotomy, with the TriGuard® cerebral protection device. The material aspirated from the left ventricle was inconclusive, and the patient underwent a six-week antibiotic regimen. No complications were reported, and follow-up showed no mass enlargement with moderate mitral regurgitation. In the second case, a 54-year-old man with vegetations on mitral and aortic bioprostheses also underwent AngioVac® thromboaspiration via transapical access. Blood reinfusion through the subclavian cannula was successful, leaving no significant residual vegetations but mild aortic regurgitation. At one-month, partial aortic prosthesis detachment and paravalvular leakage were noted, which remained stable at six months, with no new vegetations. ECMO was not needed in either case due to stable hemodynamics.

In the same year, a 57-year-old man with endocarditis on a native mitral valve presented with a large, highly mobile vegetation on the posterior leaflet, accompanied by valvular regurgitation (15). Due to a high hemorrhagic risk, thromboaspiration was selected. SpiderFX® neuroprotection filters were deployed in both femoral arteries and the right innominate artery, whilst the left subclavian artery was occluded with a balloon. AngioVac® was used for transseptal aspiration via right femoral vein access, with ECMO blood return through the left femoral vein. Nearly the entire vegetation was aspirated, leaving a small, immobile residue and persistent valvular regurgitation. The patient experienced no complications, and follow-up echocardiography revealed no new mobile vegetations and confirmed moderate valvular regurgitation.

Also reported in 2023 was a rare case involving a 72-year-old man with an atrial myxoma (21), who underwent thromboaspiration using a 22Fr AngioVac® via the transseptal approach through the right femoral vein, following the FLORIDA technique, which utilizes a loop to detach the myxoma at its base during aspiration. A Sentine®l neuroprotection device was employed, detecting no micro- thrombi. The intervention was successful, with no complications and no debris found in the Sentinel® device.

Additionally, an unusual case (14) from 2023 described a 68-year-old woman with a 26 mm mass attached to a calcified posterolateral mitral annulus. She underwent thromboaspiration via transseptal access using AngioVac®, with veno-venous access through the femoral veins. Bilateral Sentinel® devices were placed for neuroprotection. The procedure was successful without complications, and analysis of the collected fragments revealed caseous mitral annular calcification.

In 2024, a case report described a 66-year-old woman with large endocarditic vegetation on the native mitral valve, alongside a developing abscess and moderate mitral regurgitation (9). The patient was deemed high risk for surgical valve replacement because of comorbidities. Therefore, a percutaneous transseptal approach for thromboaspiration was chosen, using the AngioVac® system via the right femoral vein, with reinfusion into the left femoral vein. Sentinel® cerebral protection devices were used. The patient remained hemodynamically stable throughout the procedure and experienced no complications.

In 2024, the Lausanne Novel Procedure (4) was introduced, offering a safer approach by utilizing the Occlutech® aspiration cannula alongside triple protection of the supraaortic arteries through Sentinel® devices, effectively eliminating the need for ECMO. This technique involved a left mini- thoracotomy with transapical access to aspirate a thrombus from a mitral bioprosthesis in a 54-year- old patient. After the aspiration, micro-thrombotic residues were successfully retrieved. Remarkably, the patient experienced no complications or recurrences throughout the one-year postoperative follow-up.

The diversity of case reports highlights a significant interest in identifying the optimal methods for thromboaspiration in the left heart cavities. Nonetheless, this area remains inadequately defined, with limited consensus on the most effective techniques, aspiration devices, configurations (venous or arterial routes), and protective measures against thrombotic residues.

## 4 Discussion

### 4.1 Key types of cardiac masses of concern

Cardiac masses in the left heart cavities, ranging from benign to malignant, can significantly impact cardiac function, embolic risk, and systemic health. These masses can obstruct blood flow, invade the myocardium, or embolize, leading to a range of symptoms. While echocardiography remains the cornerstone of diagnosis, comprehensive assessment often requires multimodality imaging such as Magnetic resonance imaging (MRI) or CT scan. The differential diagnosis includes anatomical variants like pectinate muscles, thrombi, vegetations, and primary or metastatic tumors.

#### 4.1.1 Thrombus

Thrombi are the most frequent intracardiac masses (4, 16, 18, 19), commonly associated with conditions such as myocardial infarction, atrial fibrillation (22), or mitral valve disease (23). According to Gunga et al. thrombogenesis is driven by a complex interplay of hemodynamic, surface, and hemostatic factors, including low cardiac output, prosthesis malpositioning, incomplete endothelialization, leaflet damage, and hypercoagulable states like factor V Leiden (4). Inadequate anticoagulation, particularly within the first three months post-surgery, exacerbates the risk. Treatment is often surgical, with mortality rates for urgent interventions ranging from 10% to 25%. Options include valve replacement or thrombectomy. For small and recent thrombi in mild cases, intravenous heparin is the treatment of choice. Fibrinolysis, though effective in 82% of cases, has a 10% mortality rate and a 12.5% risk of systemic embolization and major bleeding (24). Contraindications include a history of intracranial hemorrhage, recent trauma, or major surgery. Despite its efficacy, fibrinolysis carries concerns over incomplete lysis or embolization, and emerging evidence supports anticoagulation as an effective treatment in many cases, reducing the need for surgery or fibrinolysis. Valve thrombosis, affecting both native and prosthetic valves (PVT), represents a significant complication. Obstructive PVT occurs in 0.3%–1.3% of mechanical valves, with thromboembolic events ranging from 0.7% to 6% per patient-year (4). For patients experiencing refractory thrombosis or those at high surgical risk, thromboaspiration provides a minimally invasive alternative.

#### 4.1.2 Vegetations

Infective vegetations, often seen on valve surfaces or abnormal flow areas or intracardiac shunts, can cause significant valvular dysfunction (9, 10, 25). Large left-sided vegetations (>10 mm) carry up to a 44% risk of embolic events, emphasizing the potential of early intervention. In the aftermath of the right sided cavities debulking with AngioVac system, percutaneous mechanical aspiration (PMA) has emerged as a promising off-label approach for managing high-risk vegetations, involving the catheter-based extraction or debulking of vegetations under imaging guidance. PMA may prevent embolic complications, reduce structural damage, and shorten antimicrobial therapy durations. It may also improve tissue sterilization and surgical outcomes by addressing infections at the source. PMA has demonstrated success in right-sided cardiac cavities but poses risks such as septic shock, septicemia, and valve destruction. Valve abscesses or severely damaged valve cusps contraindicate its use, where surgery remains the primary option altogether with systemic antibiotics. Despite these challenges, PMA offers the potential to delay or avoid surgical valve replacement, lowering prosthetic valve reinfection risks and potentially reducing hospital stays. Its inclusion in the 2023 European guidelines as a Class IIb recommendation with Level C evidence for debulking right atrial septic masses represents a significant milestone (3). There is hope that future guidelines will extend these recommendations to selected cases of mitral and aortic valve vegetations.

#### 4.1.3 Cardiac tumors

Benign cardiac tumors, including atrial myxomas and papillary fibroelastomas, are the third most common masses in cardiac cavities. Myxomas can be round or ovoid with smooth borders when encapsulated, or irregular and multilobate when gelatinous, increasing embolization risk. Papillary fibroelastomas are club-shaped with well-defined stalks. In contrast, malignant tumors are typically broad-based and multilobate, often associated with poor outcomes, while metastatic tumors vary based on their origin and site of invasion. The primary concern with cardiac tumors, aside from their histology, is their potential to obstruct blood flow. Surgical mass *in toto* resection continues to be the gold standard treatment, usually performed through sternotomy, right axillary, or thoracotomy access, utilizing cardiopulmonary bypass. Although mechanical aspiration of benign tumors is theoretically possible, the potential for recurrence due to incomplete tumor removal presents a considerable challenge. Myxomas, which are frequently anchored to the atrial septum, often require partial resection of the septum for complete removal. Notably, there is only one reported case of myxoma aspiration documented in the literature (21). The recurrence rate following mechanical aspiration remains uncertain, especially in instances where residual stalk tissue persists. In patients with recurrent atrial myxomas, such as those with Carney syndrome, percutaneous removal may be a more favorable option compared to repeated surgical interventions. Additionally, the risk of recurrence may be reduced by performing radiofrequency ablation on the myxoma stalk following tumor excision, thereby minimizing the likelihood of regrowth. Consequently, mechanical aspiration of benign tumors may be considered in highly selected patients, particularly those for whom surgical intervention poses excessive risk or is deemed unsafe.

#### 4.1.4 Other masses

Lastly, caseous mitral annulus calcification (CMAC) is an uncommon pathology that carries a heightened risk of embolic stroke and infective endocarditis (26). Due to the significant operative risks, surgical excision is rarely pursued. In a previously reported case (14), a successful removal of a CMAC mass using the AngioVac system had been illustrated.

The common thread among these masses and intracardiac elements in the left heart is their embolic potential, underscoring the critical need for a thorough exploration of neuroprotection devices.

### 4.2 The cerebral protection system (CPS)

The success and safety of thromboaspiration and intracardiac mass removal hinge on the use of cerebral embolic protection devices, which are essential for reducing the risk of cerebral embolization. The manipulation of guidewires within the left heart chambers carries the inherent risk of thrombus or mass dislodgement, which may potentially lead to significant neurological complications.

Consequently, neuroprotection in intracardiac procedures increasingly depends on devices like the Sentinel® dual filter, which has become the most widely used option in this setting. The Sentinel® system features two interconnected filters typically deployed in the brachiocephalic trunk and left common carotid artery via a trans-radial catheter (27). These filters effectively capture emboli circulating through these vessels, offering a significant advantage over deflection devices such as the TriGuard® system (28). Unlike the Sentinel® device, which captures and retrieves emboli, the TriGuard® system—currently the second most commonly used neuroprotective device—deflects emboli away from the precerebral arteries once positioned in the aortic arch. However, the main drawback of the TriGuard® is the potential for debris to embolize downstream, which poses a risk to patient outcomes.

In addition, other neuroprotection devices, such as the SpiderFX®, have been utilized in specific cases. The SpiderFX®, a nitinol filter deployed over a guidewire (29), expands into a basket that captures debris while maintaining blood flow, functioning similarly to the Sentinel as a capture device. While the literature on SpiderFX® primarily focuses on its use in femoropopliteal and peripheral vessel interventions, it has shown efficacy and non-inferiority to other devices in these contexts. Although indicated for carotid protection during angioplasty and stenting procedures, the SpiderFX® is rarely utilized for neuroprotection in routine practice and has only been mentioned twice in our review. It can be positioned exclusively in both carotid arteries (17) or used as part of a multimodal approach, whereby SpiderFX filters were placed concurrently in both superficial femoral arteries and the innominate artery, alongside a balloon in the left subclavian artery (15).

**Figure 2:**
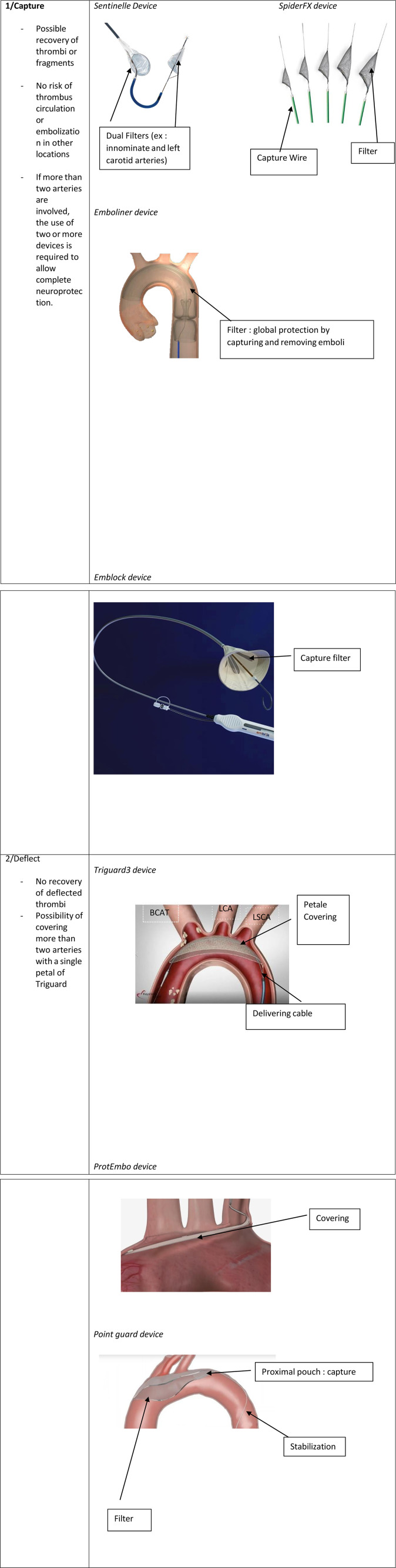
Neuroprotection devices used during thromboaspiration.

Determining the most effective neuroprotection device for thromboaspiration in left heart cavities is challenging due to several factors (Figure 2). The lack of standardization in filter placement creates variability in outcomes, complicating comparisons between devices and their positioning. Furthermore, there is a notable absence of meta-analyses addressing these filters' efficacy for this specific indication. However, evidence from percutaneous valve replacement procedures suggests a slight advantage of the Sentinel device over the Triguard® (see Table 2). A 2023 meta-analysis (30) found that the Sentinel device significantly reduces the risk of severe cerebral stroke, while the reduction associated with the Triguard® was not statistically significant. Overall, neuroprotection devices did not show statistically significant benefits for cerebral stroke and mortality. However, when evaluated individually, the Sentinel consistently yielded the best outcomes. Other studies have also highlighted the efficacy of both Sentinel and Triguard® in preventing adverse events (24, 26). A distinct advantage of the Sentinel device is its ability to capture and retrieve thrombi, eliminating the immediate embolic threat while allowing for post-procedural pathological analysis. Furthermore, the Sentinel offers the option of a radial approach, in contrast to the more invasive femoral approach required for both Triguard® and SpiderFX® (10, 15).

**Table 2:** Comparison of key cerebroprotection devices used during thromboaspiration.

Features	Sentinel®	Triguard®	SpiderFX®
Primary Function	Captures and retrieves emboli	Deflects emboli away from precerebral arteries	Captures emboli in a basket-like filter
Mechanism of Action	Dual filters capture emboli in brachiocephalic and left common carotid arteries	Deflects emboli through placement in the aortic arch	Nitinol filter deployed over a guidewire, forming a basket to trap emboli
Clinical Indication	Primarily used in percutaneous valve replacement (e.g., TAVR)	Used during procedures in the aortic arch	Indicated for carotid protection during angioplasty and stenting
Approach	Radial approach	Femoral approach	Typically femoral approach
Access Size	6Fr	8Fr	8Fr
Retrieval Capability	Yes—captures emboli for post- procedural analysis	No—deflects emboli away from target vessels	Yes—captures emboli for removal
Main Advantage	Proven to reduce risk of severe cerebral stroke	Deflects emboli without requiring retrieval	Flexible use in carotid protection and various vascular interventions
Limitations	Limited to specific arteries (brachiocephalic, left common carotid)	No retrieval of emboli; femoral approach required	Less commonly used for neuroprotection, limited literature support
Meta-analysis Findings	Significant reduction in severe cerebral stroke risk	No statistically significant reduction in severe cerebral stroke risk	Limited data for neuroprotection in cardiac procedures
Other Uses	Embolic protection during TAVR, emboli analysis post-capture	Primarily used during procedures involving the aortic arch	Occasionally used in multimodal approaches with other vascular interventions
Cases in Our Review	Most commonly used device	Second most commonly used device	Used in only two reported cases

In our review, several cases emerged where neuroprotection devices were not utilized. Notably, two cases involved the incidental formation of thrombi necessitating *in situ* aspiration (16, 19). There is only one elective case completely lacking any neuroprotection system (16). Despite the general trend toward less invasive approaches, Tsilimparis et al. (20) reported a case of thromboaspiration of a floating aortic thrombus, employing the temporary clamping of bilateral carotid arteries—of less than a minute. Despite a favorable outcome in this case, we strongly disapprove such a practice, as it is both invasive and potentially unsafe. Clamping of the carotid vessels poses a significant risk for microemboli, which can lead to adverse neurological events. An alternative approach to obstructing flow in the supraaortic arteries involves the use of a temporarily inflated balloon, such as the Armada balloon, which has been utilized in two case reports (15, 18).

In conclusion, it appears prudent to recommend the routine use of neuroprotection devices during planned aspirations of thrombi, vegetations, or cardiac masses to prevent neurological complications. While several systems have been developed, primarily for TAVI procedures, we advocate for the approach outlined in the Lausanne Novel Procedure (4), which offers near-complete protection through the deployment of two Sentinel devices: one placed in the innominate artery and left common carotid artery, and the other positioned at the entry of the left subclavian artery to also safeguard the left vertebral artery. Although this method may be time-consuming and technically demanding depending on the patient's anatomy, it allows for unimpeded blood flow and significantly reduces the neurological risk. In addition to the three devices examined in our review, emerging systems such as Point Guard®(Transverse Medical, Golden, CO, USA), Emblock®(Innovative Cardiovascular Solutions, Grand Rapids, Michigan), and ProtEmbo® (Protembis, Aachen, Germany) merit further investigation (31). These devices vary in protective strategies—either capturing or deflecting emboli—as well as access site requirements and delivery sheath sizes. As neuroprotection technologies evolve, it is crucial to thoroughly evaluate these novel devices to ensure optimal safety and efficacy in preventing cerebral embolic events during cardiac procedures. We are particularly optimistic about next-generation full-body filters like the Emboliner® (Emboline, Inc., Santa Cruz, California) and Captis® (Filterlex Medical Ltd., Caesarea, Israel), which have shown promising results in early studies (32). These devices offer comprehensive protection for both cerebral and systemic vessels, marking a significant advancement in neuroprotection technology.

### 4.3 Comparative approaches to intracardiac mass aspiration: weighing transseptal vs. transapical techniques for optimal outcomes

In the current literature, thrombi emerge as the most frequently encountered intracardiac formations, followed by vegetations and less commonly tumors. The selection of an optimal approach for the aspiration of these objects is driven by multiple factors, all aimed at balancing safety and effectiveness. Inspired by debulking techniques used in tricuspid valve endocarditis, the percutaneous route stands as the least invasive option. When it comes to accessing the left heart chambers, two primary strategies prevail: the transseptal route (purely percutaneous) and the transapical route, more invasive, which requires a mini-anterior left thoracotomy.

The choice between these approaches is largely influenced by the location of the mass and the involved valve. Typically, the transseptal approach is preferred for masses on the atrial side of the mitral valve (11, 16, 18), whereas the transapical route is more suitable for masses located in the ventricles. When addressing thrombi, the transseptal approach is generally preferred for thrombi located in atrial regions or pedunculated to the mitral valve on the atrial side. Gunga et al. (4) have described their Lausanne Novel Procedure, via a transapical aspiration for a mass which was attached to the ventricular side of a bioprosthetic mitral valve. Alternative routes such as a trans-atrial approach via the right superior pulmonary vein, have been described for the removal of thrombi from the atrial side of the mitral valve, as was reported in a 68-year-old COVID-19 patient (13). For thrombi in the aorta, both trans-caval and percutaneous femoral artery approaches are viable options (18). Ventricular thrombi, on the other hand, are most often addressed through a mini-invasive surgical approach utilizing the transapical route (4, 12). Concerning other masses, particularly tumours, aspiration is most often transseptal; however, there are greater limitations compared to thrombi or vegetations. It seems that transseptal aspiration is not feasible if the distance between the septal puncture site and the stalk is too short, or if the mass, particularly in the case of a myxoma, exceeds 2 cm, due to the low compressibility of such objects, which cannot pass through the cannula (21).

In situations where both the transseptal and transapical approaches are feasible, the decision must consider the potential complications of each approach (see Table 3). A 2023 meta-analysis (33) on the use of these approaches for mitral valve-in-valve or valve-in-ring implantation demonstrated that the transseptal approach was associated with significantly lower 30-day mortality, reduced 1-year mortality risk, and shorter hospital stays compared to the transapical approach. Although the transapical approach carries higher risks, including bleeding, pleural breaches, atrial fibrillation (34, 35) and myocardial tear, it does offer the advantage of avoiding septal puncture, thereby reducing the risk of septal defects and potential future repair (36). Another crucial consideration when selecting the optimal approach is the ability to remove the maximum, if not the entirety, of the mass while preserving the valve's function and integrity. Qintar et al. underline that a successful thromboaspiration can be considered for 70% or more removal of the thrombi or mass (18).

**Table 3:** The key differences in terms of procedural advantages and limitations of trans-septal versus trans- apical approaches.

Aspect	Transseptal Route	Transapical Access
Invasiveness	Minimally invasive, purely percutaneous approach.	More invasive, requires a mini-anterior left thoracotomy.
Access	Accesses the left atrium via the septum, suitable for atrial structure.	Direct access to the left ventricle, suitable for ventricular structures.
Patient Recovery	Shorter recovery time, associated with reduced hospital stay.	Longer recovery time due to the more invasive nature of the procedure.
Risk of Complications	Lower risk of bleeding, pleural breach, or atrial fibrillation. Potential for septal defect requiring future repair.	Higher risk of bleeding, pleural breach, and atrial fibrillation. Avoids septal puncture, reducing septal defect risk.
Effectiveness	Preferred for atrial vegetations and thrombi (e.g., mitral leaflet). May be challenging for large or rigid masses (>2 cm).	Effective for larger thrombi or masses in the ventricles.
Suitability	Best for atrial vegetations and thrombi, particularly on the atrial side of mitral leaflets	More suitable for ventricular thrombi and masses, such as ventricular side vegetations or tumors.
Complication Rates	Associated with lower 30-day and 1-year mortality according to meta-analysis	Higher rates of complications like bleeding, but no septal defects.
Technical Limitations	Limited by the size and compressibility of the mass. Difficult for rigid objects like large myxomas (>2 cm).	Suitable for larger and more rigid thrombi or masses; no size constraints related to cannula passage.
Procedure Complexity	Technically challenging due to the need for precise septal puncture.	Easier to visualize and access target structures directly, but technically demanding due to thoracotomy.
Hospital Stay	Generally shorter, quicker discharge.	Longer hospital stay due to invasive nature and recovery period (pain)
Usage in Valve Procedures	Commonly used in mitral valve-in-valve or valve-in-ring procedures, associated with better short-term outcomes	Less commonly used for valve procedures, associated with more post-operative complications.

### 4.4 The ideal aspiration canula in left sided cardiac cavities

In the realm of thromboaspiration or debulking, particularly within the left-sided cardiac cavities, the Angiovac device stands as the most widely referenced tool in the literature. Originally designed for intravascular thrombectomy and recognized as the first device capable of aspirating large thrombi and masses without the use of lytic agents (37), AngioVac has found broad off-label application in cardiac thromboaspiration. Despite its popularity and widespread use, its application is not without challenges or risks. A retrospective study published in June 2024 (38) noted the generally favorable performance of AngioVac®, but highlighted significant complications during cardiac thromboaspiration, including a case of perioperative embolization necessitating ECMO, and another where the procedure was converted to conventional surgery. Nevertheless, AngioVac® achieved approximately 80% procedural success with no per-procedural mortalities in this cohort. Other reported complications include the need for transfusions and renal impairment.

The complications associated with AngioVac®, have been further documented in a post-marketing study from the MAUDE registry in 2023 (39), which revealed several potential risks. The most frequent of these are pulmonary embolisms and vascular perforations or dissections. Less frequent but notable complications include cardiac arrests, arrhythmias, foreign body device embedment, and cardiac perforations, as evidenced by the RAPID registry. Given these risks, particularly for left-sided cardiac thromboaspiration, alternative aspiration technologies have been explored, leading to the introduction of devices like Occlutech.

Occlutech represents a softer and smaller-caliber cannula compared to AngioVac®, making it more suitable for directional thrombectomy and targeted thromboaspiration. A 2023 Swedish case report (40) emphasized the advantages of Occlutech® over Angiovac in right heart thromboaspiration. In this case, the smaller 14 Fr Occlutech® cannula outperformed AngioVac®'s larger and more rigid 22 Fr cannula. The rigidity and larger size of AngioVac®, cannulas increase the risk of vascular complications, requiring the use of two large-bore cannulas, which can lead to vessel perforation (41). Moreover, even though AngioVac®'s large catheter is designed to handle massive thrombi, its lumen can still become obstructed by large masses.

The study further suggests that Occlutech®, with its smaller diameter and more flexible cannula, reduces the risk of these complications, making it a compelling alternative to AngioVac®. Although not yet extensively used in left-sided thromboaspiration, Occlutech® is compared favorably with other advanced aspiration systems, such as the FlowTriever®, which utilizes a 16–24 Fr catheter and self-expanding nitinol discs for thrombus disruption, and the Indigo System, which also offers innovative thrombectomy capabilities.

These characteristics position Occlutech® as a preferred solution in cases where AngioVac® is either unavailable or considered too risky. Its more refined profile allows for safer maneuverability and targeted thromboaspiration in delicate areas such as the left cardiac cavities, as demonstrated in the Lausanne case. This versatility makes Occlutech® a favorable option for centers seeking a less invasive, yet highly effective, thromboaspiration device without the significant risks posed by larger, more rigid systems like AngioVac®. This shift toward Occlutech® reflects a growing trend in the field of thromboaspiration: the prioritization of both patient safety and procedural success, while minimizing complications (see Table 4).

**Table 4:** Comparative analysis of aspiration cannulas: AngioVac® versus occlutech®.

Feature	AngioVac®	Occlutech® Steerable Sheath
Design	Larger, rigid cannula (22 Fr) in a sheath of 26 Fr	Softer, smaller-caliber cannula (14 Fr)
Intended use	Initially designed for intravascular thrombectomy	To deliver left atrial appendage occluders, position ablation catheters and improves the maneuverability and placement.
Mass handling	Capable of aspirating large masses or thrombi without lytic agents	Effective for targeted thromboaspiration in delicate areas (Lausanne Novel Procedure)
Procedural success rate	Approximately 80% procedural success	Not extensively reported yet, but favorable in preliminary studies
Complications	- Pulmonary embolisms - Vascular perforations/dissections - Cardiac arrests - Need for transfusions - Renal impairment	- Reduced risk of vascular complications due to flexible design, atraumatic tip, lubricious coating and small caliber
Invasive nature	More invasive; requires two large-bore cannulas	Less invasive; can be maneuvered with a single cannula
Clinical evidence	Retrospective studies showing perioperative embolization and conversion to conventional surgery	Promising case reports indicating advantages over AngioVac®
Recovery of thrombus	Larger lumen may obstruct with massive thrombi	Smaller diameter may facilitate better retrieval of thrombotic fragments
Use in left- sided cavity	Widely referenced and commonly used from experience in right sided cavities, pulmonary embolism and intravascular aspiration	Emerging use; potential for wider application in left-sided thromboaspiration
Suitability	Best for patients requiring rapid, large thrombus removal	Ideal for patients with delicate anatomical considerations and less invasive needs

### 4.5 Comprehensive techniques, setup, and procedural insights

#### 4.5.1 Percutaneous transeptal approach

**Figure 3:**
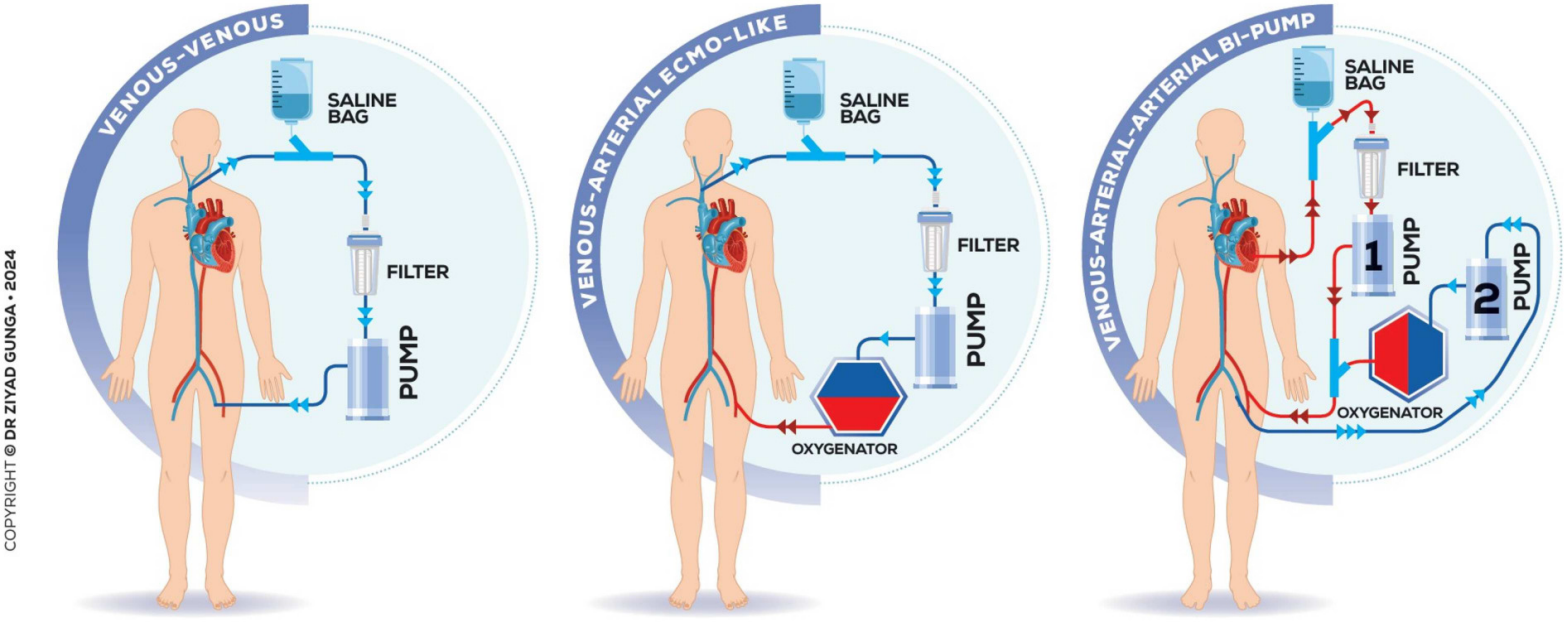
Various configurations and extracorporeal circuits of the technique.

The thromboaspiration technique via the transseptal approach mostly utilizes the third-generation 180° 24 Fr AngioVac catheter combined with an extracorporeal bypass circuit and re-infusion cannula, requiring two access points for aspiration and reperfusion. The bypass circuit includes an outflow line to the AngioVac cannula, a centrifugal pump, a filter, and an inflow line to the return cannula. Activating the pump generates unidirectional flow, creating suction at the catheter tip to draw and filter blood, which is then reinfused to minimize blood loss. For left-sided lesions, either arteriovenous (AV), arterio-arterial (AA) and veno-venous configurations are available to accommodate anatomical variations (Figure 3). Advancing the AngioVac system through the left atrium after transseptal access can be challenging, especially with a thickened septum. The process often results in blood loss when removing the dilator. To mitigate these issues, we advocate for the balloon-assisted tracking technique described by Quintar et al. In this method, a 10 mm Armada balloon is preloaded in the AngioVac catheter over a wire, inflated outside the body, and advanced as a single unit toward the septum. Once at the septum, the balloon is deflated and used to dilate it, allowing smooth introduction of the AngioVac into the left atrium. This technique not only reduces blood loss but also enhances tracking and device advancement. If ECMO is employed, the outflow cannula is placed in series with the ECMO's venous cannula (Figure 3).

#### 4.5.2 Transapical approach via a left mini-invasive thoracotomy

**Figure 4:**
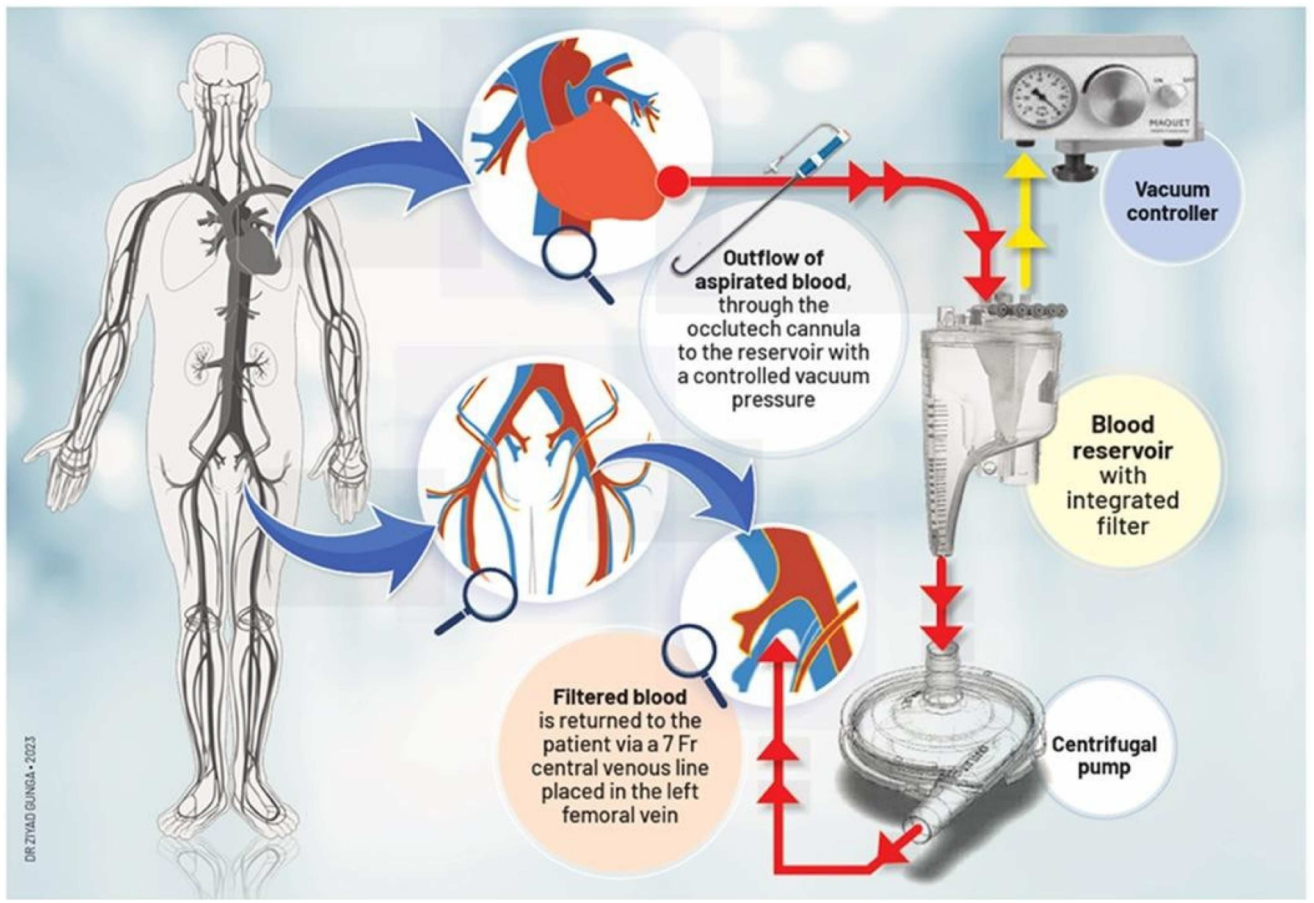
Description of thromboaspiration by a trans-apical access using the occlutech canula.

The patient is positioned supine with a roller pad under the left scapula to provide a slight rightward tilt and is intubated with a double-lumen tube. The left radial artery and groins are prepared within the sterile field. A 4 cm transverse incision is made 1 cm below the areolar line, directed toward the apex identified via echocardiography. The fifth intercostal space is accessed, confirming no lung adhesions. A soft tissue and rib spreader retractor are placed, and the pericardium is opened over 4 cm, showing minimal adhesion. Stay sutures allow clear apex exposure, pinpointed by transesophageal echocardiography (TEE). Two concentric purse-string sutures are placed with Ethibond-3/0 and PTFE-felt pledgets, securing bites through the muscle without entering the left ventricular cavity. Hemodynamics are closely monitored, and TEE and fluoroscopy guide the next steps. The apex is punctured, and a soft guidewire is inserted across the aortic valve, avoiding the thrombus. A 14 Fr Occlutech steerable guiding sheath is inserted and connected to a pediatric extracorporeal circuit with a reservoir, filter, centrifugal pump, and reinjection catheter via the left femoral vein (Figure 4). The Occlutech tip is guided into the LVOT, aspirating the thrombus and filtering and reinfusing the blood back to the patient via the femoral vein, without the use of concomitant ECMO. Alternatively, the AngioVac system can be utilized in place of the Occlutech cannula for thrombus aspiration.

#### 4.5.3 Optimal use of ECMO in thromboaspiration: weighing the benefits and risks

The decision of whether to use ECMO during thromboaspiration procedures is a critical one, requiring careful consideration of both benefits and risks. The literature highlights several cases where concomitant ECMO has been employed during thromboaspiration (12, 13, 15), especially in elderly or high-risk patients. ECMO, particularly the veno-venous and more invasive arterio-venous configurations, offers substantial circulatory and respiratory support, stabilizing patients hemodynamically during intraoperative and postoperative phases. This can be advantageous in those with hemodynamic instability, shock, or respiratory failure. However, ECMO use introduces significant complications that cannot be overlooked.

Key risks associated with ECMO include increased platelet aggregation, reduction of factor VIII and von Willebrand factor, and decreased fibrinogen levels within the first 24 h (42). In addition, ECMO is associated with a higher likelihood of bleeding, thromboembolic events—including intracardiac thrombi in arterio-venous ECMO (43)—air embolism, and mechanical complications such as circuit cavitation, limb ischemia, vessel perforation, dissection, and pseudoaneurysms (44). The risk of neurological complications, such as ischemic stroke, hypoxic-ischemic brain injury, and brain death, is also elevated (45), particularly in arterio-venous configurations.

One specific drawback during the thromboaspiration phase is that the patient's preload must be adequately maintained, and the duration of aspiration kept short. In fact, rather than focusing purely on the time of aspiration, it is the volume aspirated that poses the greatest challenge. Aspiring too much volume can depress the patient's hemodynamic stability, risking circulatory collapse. Gerosa et al. reported the necessity of using concomitant ECMO in their cases to prevent such hemodynamic deterioration (13), especially during prolonged aspiration phases, where the circulatory demand could not be supported otherwise.

Recent trends in thromboaspiration, however, indicate a growing preference for avoiding concomitant ECMO, even when using sophisticated systems like AngioVac®. This approach is particularly relevant for patients who are hemodynamically stable (10), without shock, or those without significant risk factors for intraoperative or postoperative hemodynamic collapse. Our literature review identified nearly 20 case reports where ECMO was successfully avoided (9–11, 14, 17, 18), with the thromboaspiration circuit—utilizing filtration and blood reinfusion—acting as a simple extracorporeal circulation (ECC), proving sufficient for stable patients.

In our practice, we advocate for minimizing the use of ECMO when feasible. The Lausanne thromboaspiration technique specifically avoids concomitant ECMO, reserving its use only for cases where the patient is in shock or faces a severe risk of intraoperative or postoperative hemodynamic instability. The primary goal of thromboaspiration is to remain minimally invasive, and the use of ECMO—while sometimes lifesaving—adds complexity and invasiveness to the procedure.

From a medico-economic perspective, avoiding ECMO when feasible is prudent, as it incurs significant procedural costs (46) and potential financial burdens from associated complications (47). Methods that reduce or eliminate ECMO during thromboaspiration align with the procedure's minimally invasive nature and offer substantial cost benefits. Ultimately, ECMO should be reserved for cases where its benefits clearly outweigh the risks, ensuring that overall invasiveness and costs remain low while maintaining patient safety.

#### 4.5.4 Optimizing patient selection and a multidisciplinary approach

The best candidates for left-sided mass aspiration are carefully selected based on clinical factors such as the nature of the mass, its location, and the patient's overall risk profile. Ideal candidates include those with intracardiac thrombi resistant to anticoagulation therapy, where surgery poses high risks. Thromboaspiration offers a less invasive alternative, particularly for patients with mobile or pedunculated masses, such as thrombi or vegetations attached to cardiac structures like valves, which carry a high embolic risk. Moderate-sized masses (less than 2–3 cm), especially non-calcified and friable thrombi, are generally more amenable to this approach. Additionally, patients with large left-sided vegetations (>10 mm) at risk of embolic events and not suited for immediate surgery can benefit from aspiration to reduce embolic potential and stabilize infections.

A multidisciplinary team is essential for managing these patients, involving cardiac surgeons, cardiologists, interventional cardiologists, echographers, and anesthetists. Collaborative discussions ensure comprehensive preoperative evaluations, utilizing advanced imaging techniques like transesophageal echocardiography (TEE) and fluoroscopy to guide procedures and prevent complications. TEE enables real-time visualization of cardiac structures, facilitating careful maneuvering without dislodging masses or damaging sensitive areas like the septum, mitral valve, or chordae tendineae. Additionally, careful monitoring of anticoagulation is critical, with an activated clotting time (ACT) threshold varying from 180 (13) to 300 (9) seconds recommended to reduce bleeding risks. While surgical resection is the gold standard for most tumors and heavily calcified masses, thromboaspiration offers a safer, less invasive alternative for select patients, minimizing complications and improving outcomes.

## 5 Conclusion

In conclusion, left-sided thromboaspiration is emerging as a safe, reproducible, and minimally invasive technique with low complication rates. It offers significant potential not only for critically ill or surgically ineligible patients but also as a primary approach for managing thrombi. The involvement of a multidisciplinary team enhances its success, making it a versatile option for broader use. While formal guidelines have yet to be established, there is optimism that left-sided thromboaspiration will soon be recognized as a valuable procedure in routine practice. Our review aimed to provide all the necessary insights and critical judgment on current practices, emphasizing the strengths and areas for improvement. As this field continues to evolve, left-sided thromboaspiration has the potential to reshape the landscape of minimally invasive cardiac care.
